# Effectiveness of intramuscular corticosteroid injection versus placebo injection in patients with hip osteoarthritis: design of a randomized double-blinded controlled trial

**DOI:** 10.1186/1471-2474-12-280

**Published:** 2011-12-12

**Authors:** Desirée MJ Dorleijn, Pim AJ Luijsterburg, Max Reijman, Margreet Kloppenburg, Jan AN Verhaar, Patrick JE Bindels, P Koen Bos, Sita MA Bierma-Zeinstra

**Affiliations:** 1 Department of General Practice, Erasmus MC, University Medical Center, Rotterdam, PO Box 2040, 3000 CA Rotterdam, The Netherlands; 2 Department of Orthopaedics, Erasmus MC, University Medical Center, Rotterdam, PO Box 2040, 3000 CA Rotterdam, The Netherlands; 3 Department of Rheumatology, LUMC, PO Box 9600, 2300 RC Leiden, The Netherlands

## Abstract

**Background:**

Recent international guidelines recommend intra-articular corticosteroid injections for patients with hip osteoarthritis who have moderate to severe pain and do not respond satisfactorily to oral analgesic/anti-inflammatory agents. Of the five available randomized controlled trials, four showed positive effects with respect to pain reduction. However, intra-articular injection in the hip is complex because the joint is adjacent to important neurovascular structures and cannot be palpated. Therefore fluoroscopic or ultrasound guidance is needed.

The systemic effect of corticosteroids has been studied in patients with impingement shoulder pain. Gluteal corticosteroid injection was almost as effective as ultrasound-guided subacromial corticosteroid injection. Such a clinically relevant effect of a systemic corticosteroid injection offers a less complex alternative for treatment of patients with hip osteoarthritis not responsive to oral pain medication.

**Methods/Design:**

This is a double-blinded, randomized controlled trial. A total of 135 patients (aged > 40 years) with hip osteoarthritis and persistent pain despite oral analgesics visiting a general practitioner or orthopaedic surgeon will be included. They will be randomized to a gluteal intramuscular corticosteroid injection or a gluteal intramuscular placebo (saline) injection. The randomization will be stratified for setting (general practitioner and outpatient clinics of department of orthopaedics). Treatment effect will be evaluated by questionnaires at 2, 4, 6, and 12 weeks follow-up and a physical examination at 12 weeks. Primary outcome is severity of hip pain reported by the patients at 2-week follow-up. Statistical analyses will be based on the intention-to-treat principle.

**Discussion:**

This study will evaluate the effectiveness of an intramuscular corticosteroid injection on pain in patients with hip osteoarthritis. Patient recruitment has started.

**Trial Registration:**

This trial is registered in the Dutch Trial Registry: number NTR2966.

## Background

Recent international guidelines recommend intra-articular (IA) corticosteroid injections for patients with hip osteoarthritis (OA) who have moderate to severe pain and no satisfactory response to oral analgesic/anti-inflammatory agents [1]. Of the five randomized controlled trials (RCTs) on this subject [2-6], four showed clinically significant positive effects with respect to pain reduction (effect size up to 1.5 at 1 week follow-up) [2,4-6] and one showed no clinical benefit of an IA injection [3]. In the RCT that showed no clinical benefit of an IA injection, patients were biased towards a negative result having been informed they would receive priority for surgery if their pain worsened after injection [3].

Because the hip joint is adjacent to important neurovascular structures and cannot be palpated, IA injection under fluoroscopic or ultrasound guidance is advised. However, these techniques are not always available, especially in a primary care setting. Moreover, apart from being complex, an IA hip injection can be painful for the patient and can lead to septic arthritis. An effective but simpler administration technique would be a welcome addition to the current methods to treat episodes of increased pain in hip OA.

A double-blind RCT in patients with subacromial impingement shoulder pain showed almost equal effectiveness of ultrasound-guided subacromial corticosteroid injection compared to gluteal (systemic) injection [7]; this effect was also reported in an earlier study [8]. In addition, an equal or even more pronounced pain decrease was found in patients with concurrent hip OA or chronic low back pain in an RCT assessing the effectiveness of a local corticosteroid injection in patients with greater trochanteric pain syndrome [9,10]. These results indicate a systemic effect of corticosteroids on pain in OA.

A clinically relevant effect of a systemic corticosteroid injection, offers a less complex alternative for treatment of patients with hip OA who are not responsive to oral pain medication. Since IA hip injection is not standard care in the Netherlands, we decided to conduct a trial comparing intramuscular (IM) corticosteroid injection versus IM placebo injection.

### Primary objective

This RCT will assess the effectiveness of an IM gluteal corticosteroid injection versus an IM gluteal placebo injection for pain in patients with hip OA who have moderate to severe pain and no satisfactory response to oral analgesic/anti-inflammatory agents during 12-weeks follow-up.

### Secondary objectives

The study will assess the effectiveness of an IM gluteal corticosteroid injection versus an IM gluteal placebo injection in patients with hip OA with regard to function, mobility and patients' perceived improvement. Adverse reactions will be registered and an explorative subgroup analysis will be performed stratified for setting (general practitioner and outpatient clinics of department of orthopaedics).

## Methods

### Design

This is a double-blinded RCT. The Medical Ethics Committee of the Erasmus University Medical Center approved the trial (MEC2011-115). All patients will provide written informed consent.

### Patient selection

Patients with hip OA will be recruited in primary care (general practices in the Rotterdam area) and via hospital referrals (orthopaedic outpatient clinics in the Rotterdam area). Treating physicians are asked to select patients with hip OA and screen them on the inclusion/exclusion criteria (Table 1). If a patient has bilateral hip OA, the most painful hip will be selected as the study hip.

**Table 1:** Patients' eligibility criteria

	Inclusion criteria:
1.	Hip OA* according to clinical ACR** criteria
2.	Age > 40 years
3.	Symptomatic disease for at least 6 months prior to enrolment
4.	Radiographic evidence of OA* (Kellgren-Lawrence score ≥ 2)
5.	Persistent pain despite receiving optimal doses of oral pain medication for at least 3 weeks. Pain severity (in rest or on walking) defined as ≥ 3 on an NRS^# ^(0-10 range, 0 = no pain)
	**Exclusion criteria:**
1.	Inability to understand Dutch questionnaires
2.	Systemic infection
3.	Local infection
4.	Systemic arthritis
5.	Diabetes mellitus
6.	Coagulopathy
7.	Gastric ulcer
8.	Current use of oral corticosteroids, DMARDs^$ ^or immunosuppressive medication
9.	Allergy to corticosteroids
10.	Anticoagulant therapy (coumarins)
11.	On the waiting list for total hip replacement surgery
12.	IA^## ^injection into the hip in the previous 6 months
13.	Radiologic signs of osteonecrosis
14.	Participation in other medical trial
15.	Pregnancy or lactating female

* *OA *Osteoarthritis; **ACR American College of Rheumatology; ^# ^*NRS *Numerical Rating Scale; $ *DMARD *disease-modifying antirheumatic drugs; ^## ^*IA *intra-articular

### Procedures

Eligible patients will receive written study information from their treating physician. If they show interest, the physician will fax their contact data to the research team. The researcher will contact the patient to answer additional questions. If the patient is interested/willing to participate, an appointment at the research centre will be made to sign an informed consent form and screen on inclusion/exclusion criteria, including assessment of radiologic hip OA. Pelvic anteroposterior (AP) X-rays taken within 6 months prior to enrolment are accepted; otherwise an AP pelvic X-ray will be taken. Two researchers will independently of each other assess grading of hip OA according to Kellgren-Lawrence (K&L) [11]. If the patient meets the radiologic criteria for participation (K&L score of ≥ 2), baseline measurement (questionnaire and physical examination) follows.

### Randomisation

An independent pharmacy assistant will allocate each patient based on computerized randomization lists to either receive placebo (saline) injection or triamcinolone acetate 40 mg injection IM. Randomization is stratified for setting (general practitioner and outpatient clinics of department of orthopaedics) and uses random blocks of 2 and 4.

### Blinding

To assure blinding with respect to the patient, researcher and treating physician, the trial medication will be packed and sealed by the pharmacy of the Erasmus MC, Rotterdam. An independent research assistant (who is not otherwise involved in the study) will prepare and administer, out of sight of the patient, the injection in the upper lateral quadrant of the gluteal musculature. The injection will be administered in the gluteal area ipsilateral of the study hip.

### Intervention

Patients who participate in the trial are randomized to either an IM triamcinolone acetate 40 mg injection once or an IM saline injection once.

Patients are allowed to continue their usual pain medication or physical therapy, but are requested not to start any new therapies regarding their hip OA during study follow-up.

### Outcomes

#### Questionnaires at baseline, 2, 4, 6 and 12 weeks

All outcomes are measured at baseline and at 2, 4, 6 and 12 weeks follow-up. The primary outcome is severity of hip pain reported by the patient at 2 weeks. This will be measured with two validated questionnaires: an 11-point numerical rating scale (NRS) in rest and on walking (0-10, where 0 equals no pain) [12], and the Western Ontario and McMaster University Osteoarthritis Index (WOMAC) pain subscale [13]. The WOMAC pain subscale will be converted to a 0-100 score, where 0 equals no symptoms. The WOMAC is recommended by the Osteoarthritis Research Society for use in clinical trials in patients with hip OA to measure pain and disability [13].

Secondary outcomes include the primary outcomes at 4, 6, and 12 weeks follow-up. Additional secondary outcomes are the disease-specific WOMAC function and stiffness subscales [13]. Both the function and stiffness subscale of the WOMAC will be converted to a 0-100 score. Data on pain and function will also be obtained with the Hip disability and Osteoarthritis Outcome Score (HOOS) [14], which was developed as an extended version of the WOMAC to evaluate the whole domain of patient-relevant outcome in young and active patients and is validated in the Dutch language [15].

For patients' perceived recovery a 7-point Likert scale will be used (score range 1 = 'worse than ever' to 7 = 'major improvement') [9,16]. Quality of life will be measured with the Euroqol (EQ-5D) [17]. Constant and intermittent pain will be obtained with the questionnaire Intermittent and Constant Osteoarthritis Pain (ICOAP) taking into account both pain intensity and impact on quality of life [18]. Patients' medical consumption will be registered and adverse reactions noted [9,16]. For this, a questionnaire will be used that covers the known local and systemic adverse reactions to corticosteroids. Data on daily pain and pain medication use will be obtained with a diary during the first 2-weeks follow-up.

Another secondary outcome is the difference in percentage of responders as defined by the OMERACT-OARSI (improvement in at least 2 of the 3 following domains: ≥20% improvement in WOMAC pain, ≥20% improvement in WOMAC function, or markedly improved on the patients' global assessment) [17].

At baseline various patient characteristics (gender, age, height, weight, race, education, marital status, occupational situation and co-morbidities) are recorded. Table 2 presents an overview of the parameters measured during follow-up.

**Table 2:** Timing of measurements and outline of primary and secondary outcome measures

	Baseline	Daily diary for 2 weeks	2 weeks	4 weeks	6 weeks	12 weeks
**Primary outcome measures**						

Pain score (WOMAC*)	x		x	x	x	x

Pain Score (NRS**)	x	x	x	x	x	x

**Secondary outcome measures**						

Function score (WOMAC)	x		x	x	x	x

Stiffness score (WOMAC)	x		x	x	x	x

HOOS***	x		x	x	x	x

Quality of life (EuroQol EQ-5D)	x		x	x	x	x

Constant and intermittent pain (ICOAP^#^)	x		x	x	x	x

Use of medication	x	x	x	x	x	x

Medical consumption	x		x	x	x	x

Adverse reactions			x	x	x	x

Perceived recovery			x	x	x	x

**Others**						

Demographic data	x					

Co-morbidity	x					

Physical examination hip, knee and lumbar spine	x					x

Laboratory assessment (ESR^##^, Hs-CRP^###^)	x					

* *WOMAC *Western Ontario and McMaster University Osteoarthritis Index

** *NRS *Numerical Rating Score

*** *HOOS *Hip disability and Osteoarthritis Outcome Score

^# ^*ICOAP *Intermittent and Constant Osteoarthritis Pain

^## ^*ESR *Erythrocyte Sedimentation Rate

^### ^*Hs-CRP *high sensitive C-reactive protein

#### Physical examination at baseline and 12-weeks follow-up

Both hip joints will be examined for presence of groin pain or peri-trochanteric pain at palpation, range of motion and pain at/during movement for flexion/extension, abduction, and for internal/external rotation. Hip rotations will be examined in sitting position with the hips and knees in 90°. Hip flexion/extension and abduction will be examined in supine position. A goniometer will be used to measure degrees of range of motion [19].

To gain insight in knee and lumbar spine co-morbidity both knee joints and lumbar spine will be examined. Pain at palpation of the medial or lateral joint space of the knee, hydrops and range of motion of flexion/extension of the knee will be registered. Pain at palpation of the spinous processes or sacro-iliac joints and lateroflexion and flexion of the lower spine (fingertip-floor distance and classic Schober test) will be examined [20].

#### Laboratory assessment

At baseline, two blood samples (9 ml) will be collected. One to measure the erythocyte sedimentation rate, which is used for the American College of Rheumatology criteria of hip OA [21]. The other for high-sensitive C-reactive protein (hs-CRP) to gain insight in the inflammatory processes. The samples will be analysed at the Trial Laboratory Department of the Erasmus MC.

### Sample size

Data from the Qvistgaard et al. study (patients with hip OA from primary care and secondary care) were used to calculate our sample size [6]. That study showed a baseline standard deviation (SD) of 20 for pain at rest and at walking (0-100 visual analogue scale; VAS). Assuming a minimal clinically relevant difference of 10 points (effect size 0.5), 64 patients per group will be needed to show a statistically significantly difference using 80% power and with a 5% alpha.

In that same study [6] the WOMAC total score (0-96) was used with an SD of 15. Standardized to a 0-100 score this SD is almost 16. Assuming an SD of 16 and an 8-point difference as clinically relevant (effect size 0.5), the same sample size is needed.

We checked these scores in a Dutch study population with hip OA, i.e. those with a K-L score of the hip ≥ 2 and a VAS pain score ≥ 30, participating in the GOAL study [22]. This showed they had a mean VAS score of 56.4 with an SD of 19.3, a mean WOMAC pain score of 51.2 with a SD of 16.4; these data are very similar to the SDs in the study of Qvistgaard et al. Therefore, in the planned trial, we will include 135 patients, anticipating only 5% loss to follow-up based on the relatively short follow-up and earlier experience with loss to follow-up [22,23].

### Data analyses

Data analysis will be performed based on the 'intention to treat' principle.

Descriptive statistics will be used to describe patient's characteristics, items of physical examination, and the severity of radiologic hip OA.

Linear mixed models will be used for repeated measures to analyze the continuous outcome measures. Fixed effects will be time, time by therapy and the covariates we adjust for. For patients lost to follow-up, we will include all observed data in the analysis. Adjustment will be made for those baseline variables that change the effect estimate by more than 10%. Similar analyses with Generalized Estimating Equations techniques for repeated measures will be done for dichotomous outcome measures.

Subgroup analyses for setting will be analyzed by assessing interaction effects between type of intervention and setting on the primary outcomes; in addition, the estimates will be shown for both settings separately. We realize that these subgroup analyses will remain solely explorative because our sample size is not directed to powerful subgroup analyses.

## Competing interests

The authors declare that they have no competing interests.

## Authors' contributions

SMABZ, JANV, MK, MR, PKB, PJEB and PAJL participated in the design and coordination of the study. DMJD coordinates the trial and is responsible for data acquisition. DMJD and PAJL prepared the article. All authors have read and approved the final manuscript.

## Pre-publication history

The pre-publication history for this paper can be accessed here:

http://www.biomedcentral.com/1471-2474/12/280/prepub
